# Vaccine decision-making among pregnant women: a protocol for a cross-sectional mixed-method study in Brazil, Ghana, Kenya and Pakistan

**DOI:** 10.12688/gatesopenres.16280.1

**Published:** 2024-08-29

**Authors:** Jessica L Schue, Prachi Singh, Berhaun Fesshaye, Emily S Miller, Shanelle Quinn, Ruth A Karron, Renato T Souza, Maria Laura Costa, Jose Guilherme Cecatti, Kwasi Torpey, Caroline Dinam Badzi, Emefa Modey, Chris Guure, Ferdinand Okwaro, Marleen Temmerman, Saleem Jessani, Sarah Saleem, Muhammad Asim, Sidrah Nausheen, Haleema Yasmeen, Grace Belayneh, Vanessa Brizuela, Sami Gottlieb, Rupali J Limaye

**Affiliations:** 1 Department of International Health, Johns Hopkins Bloomberg School of Public Health, Baltimore, Maryland, USA; 2 Department of Obstetrics and Gynecology, University of Campinas, Campinas, State of São Paulo, Brazil; 3 Department of Population, Family and Reproductive Health, School of Public Health, University of Ghana, Accra, Greater Accra Region, Ghana; 4 Department of Maternal and Child Health, School of Nursing and Midwifery, University of Ghana, Accra, Greater Accra Region, Ghana; 5 Department of Biostatistics, School of Public Health, University of Ghana, Accra, Greater Accra Region, Ghana; 6 Centre of Excellence in Women and Child Health, Aga Khan University, Nairobi, Nairobi County, Kenya; 7 Aga Khan University, Karachi, Sindh, Pakistan; 8 Jinnah Post Graduate Medical Centre, Karachi, Sindh, Pakistan; 9 UNDP/UNFPA/UNICEF/WHO/World Bank Special Programme of Research, Development and Research Training in Human Reproduction, Department of Sexual and Reproductive Health and Research, World Health Organization, Geneva, Switzerland

**Keywords:** COVID-19, pregnancy, maternal immunization, Brazil, Ghana, Kenya, Pakistan

## Abstract

Maternal immunization is a critical strategy to prevent both maternal and infant morbidity and mortality from several infectious diseases. When the first COVID-19 vaccines became available during the pandemic, there was mixed messaging and confusion amongst the broader public and among those associated with health care systems about the recommendations for COVID-19 vaccinations in pregnancy in many countries. A multi-country, mixed-methods study is being undertaken to describe how vaccine decision-making occurs amongst pregnant and postpartum women, with a focus on COVID-19 vaccines. The study is being conducted in Brazil, Ghana, Kenya, and Pakistan. In each country, participants are being recruited from either 2 or 3 maternity hospitals and/or clinics that represent a diverse population in terms of socio-economic and urban/rural status. Data collection includes cross-sectional surveys in pregnant women and semi-structured in-depth interviews with both pregnant and postpartum women. The instruments were designed to identify attitudinal, behavioral, and social correlates of vaccine uptake during and after pregnancy, including the decision-making process related to COVID-19 vaccines, and constructs such as risk perception, self-efficacy, vaccine intentions, and social norms. The aim is to recruit 400 participants for the survey and 50 for the interviews in each country. Qualitative data will be analyzed using a grounded theory approach. Quantitative data will be analyzed using descriptive statistics, latent variable analysis, and prediction modelling. Both the quantitative and qualitative data will be used to explore differences in attitudes and behaviors around maternal immunization across pregnancy trimesters and the postpartum period among and within countries. Each country has planned dissemination activities to share the study findings with relevant stakeholders in the communities from which the data is collected and to conduct country-specific secondary analyses.

## Introduction

Vaccination during pregnancy can be recommended for a variety of reasons: to prevent disease in the pregnant woman, to protect the fetus and prevent pregnancy complications, and to decrease morbidity and mortality in women, newborns and infants. Maternal immunization can compensate for newborns’ inexperienced immune systems, by allowing the mother to transmit protective antibodies to her baby via the placenta or breast milk ( Röbl-Mathieu *et al.*, 2021). Additionally, antibodies transferred from parent to child either during pregnancy or after childbirth play a crucial role in decreasing morbidity and mortality in newborns and infants ( Marchant *et al.*, 2017). In the case of COVID-19, a meta-analysis found that immunization reduces the risk of hypertensive disorders in pregnancy, reduces the likelihood of caesarean section, and reduces a newborn’s risk of being admitted to the neonatal intensive care unit ( Fernández-García *et al.*, 2024). Vaccinating pregnant women is currently recommended for a variety of diseases, including tetanus, pertussis, influenza, hepatitis B and COVID-19, and additional maternal vaccines are expected to be introduced in the coming years ( Geoghegan *et al.*, 2022; Limaye *et al.*, 2024). Maternal vaccines can serve as a crucial prevention tool for common diseases in infancy, such as Group B streptococcus, where currently available screening and/or treatment are complex and may be further challenged by health system constraints, or where births frequently occur outside of health facilities ( Rao & Khanna, 2020).

However, despite the congruence of evidence and policies supporting the safety and benefits of several maternal vaccines, there remains considerable disparity in their use and coverage both among and within countries ( Laenen *et al.*, 2015; Sobanjo-ter Meulen *et al.*, 2019). Attitudes and decision-making regarding maternal immunizations are complex; pregnant women must weigh the risk-benefit ratio for both themselves and their fetus ( Cox *et al.*, 2023). There are a multitude of factors that influence maternal immunization decision-making, among these are the opinions and recommendations of family and healthcare providers ( Cox *et al.*, 2023; Kilich *et al.*, 2020; Limaye *et al.*, 2022). Immunization decision-making while pregnant and in the postpartum period is also influenced by other factors, such as risk perception, knowledge of the disease and vaccine, social norms, and self-efficacy, to name a few ( Cox *et al.*, 2023; Kilich *et al.*, 2020).

During the height of the pandemic, pregnant women with COVID-19 were shown to be at greater risk of severe disease, hospital admission, and pre-term birth ( Allotey *et al.*, 2020; Smith *et al.*, 2023). But with the exclusion of pregnant individuals from the vast majority of COVID-19 vaccine trials, there was limited early vaccine safety data for this population and large variation in countries’ initial policy recommendations for COVID-19 vaccine use in pregnancy ( Hameed *et al.*, 2023; Zavala *et al.*, 2022). Over time, the availability of additional vaccine safety and effectiveness data for pregnant women led to more countries recommending or permitting the use of COVID-19 vaccines during pregnancy ( Hameed *et al.*, 2023; Prasad *et al.*, 2022; Wang *et al.*, 2022; Zavala *et al.*, 2022). But these varying and changing policies gave considerable latitude in the way local advisory groups and managers interpreted vaccine recommendations. The World Health Organization (WHO) now recommends a dose of COVID-19 vaccine to be given during each pregnancy ( World Health Organization, 2023). However, even where COVID-19 vaccination during pregnancy has been strongly encouraged, uptake has been sluggish ( Blakeway *et al.*, 2022; Goncu Ayhan *et al.*, 2021; Razzaghi *et al.*, 2021; Shamshirsaz *et al.*, 2022).

**Figure 1.**
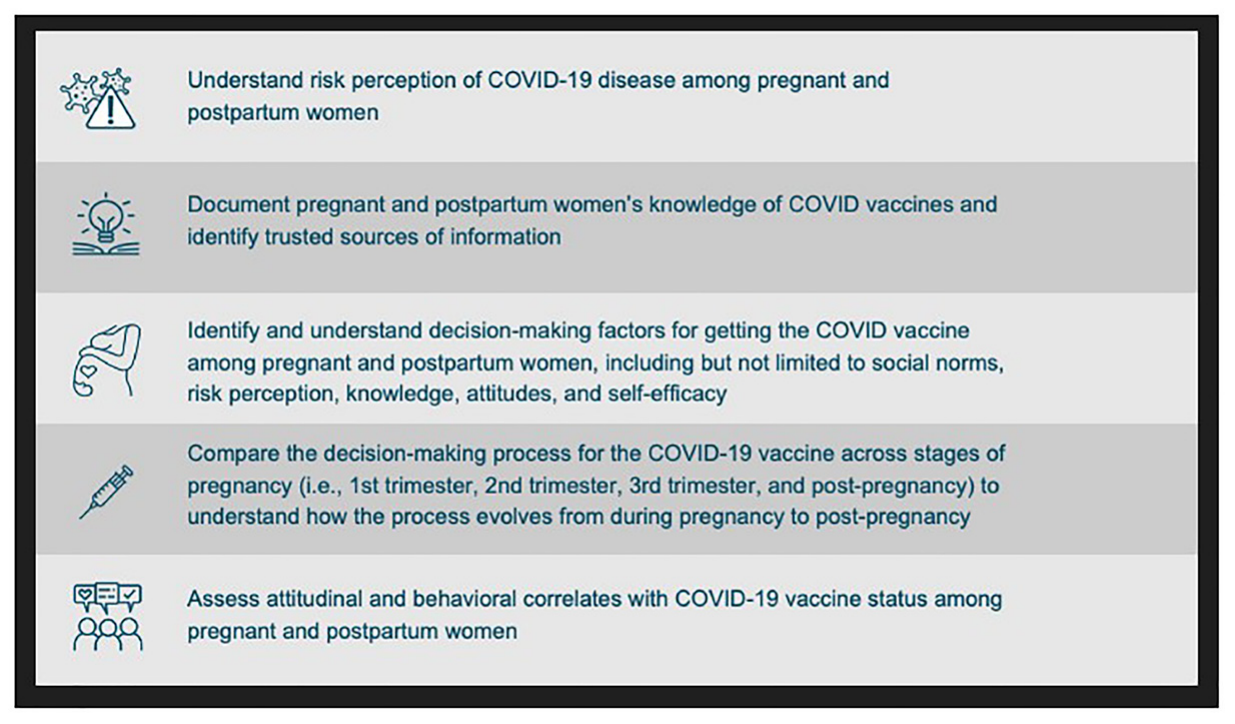
Objectives for a multi-country, mixed methods, cross-sectional study.

To better inform demand generation and communication strategies for vaccines in pregnancy, it is crucial to address several knowledge gaps and gather information from pregnant and postpartum women to understand factors that influence their vaccine decision-making process. This paper describes the protocol and early implementation for a mixed methods study to better understand how COVID-19 vaccine decision-making occurs, including attitudes about maternal immunization more broadly, among pregnant and postpartum women in Brazil, Ghana, Kenya, and Pakistan. The study includes five objectives ( Figure 1) with an aim to strengthen guidance, policy, and programs related to COVID-19 vaccination of pregnant women, especially in low- and middle-income countries.

## Methods

### Study design

This descriptive study aims to understand COVID-19 vaccine decision-making amongst pregnant women. The study objectives are being addressed using mixed methods across four countries consisting of cross-sectional quantitative surveys among pregnant women and qualitative semi-structured in-depth interviews with both pregnant and postpartum women. The multi-country study team consists of an interdisciplinary group of researchers and policy makers with expertise in vaccine and behavioral science, obstetrics and nursing, maternal and child health, epidemiology, and biostatistics, as well as mixed method study design and data collection expertise in both quantitative and qualitative methods.

This document uses the term ‘pregnant women’. Although most people who are or can get pregnant are cisgender women who were born and identify as female, these topics are also relevant to the experiences of transgender men and other gender diverse people who may have the capacity to become pregnant.

### Study locations

**Figure 2.**
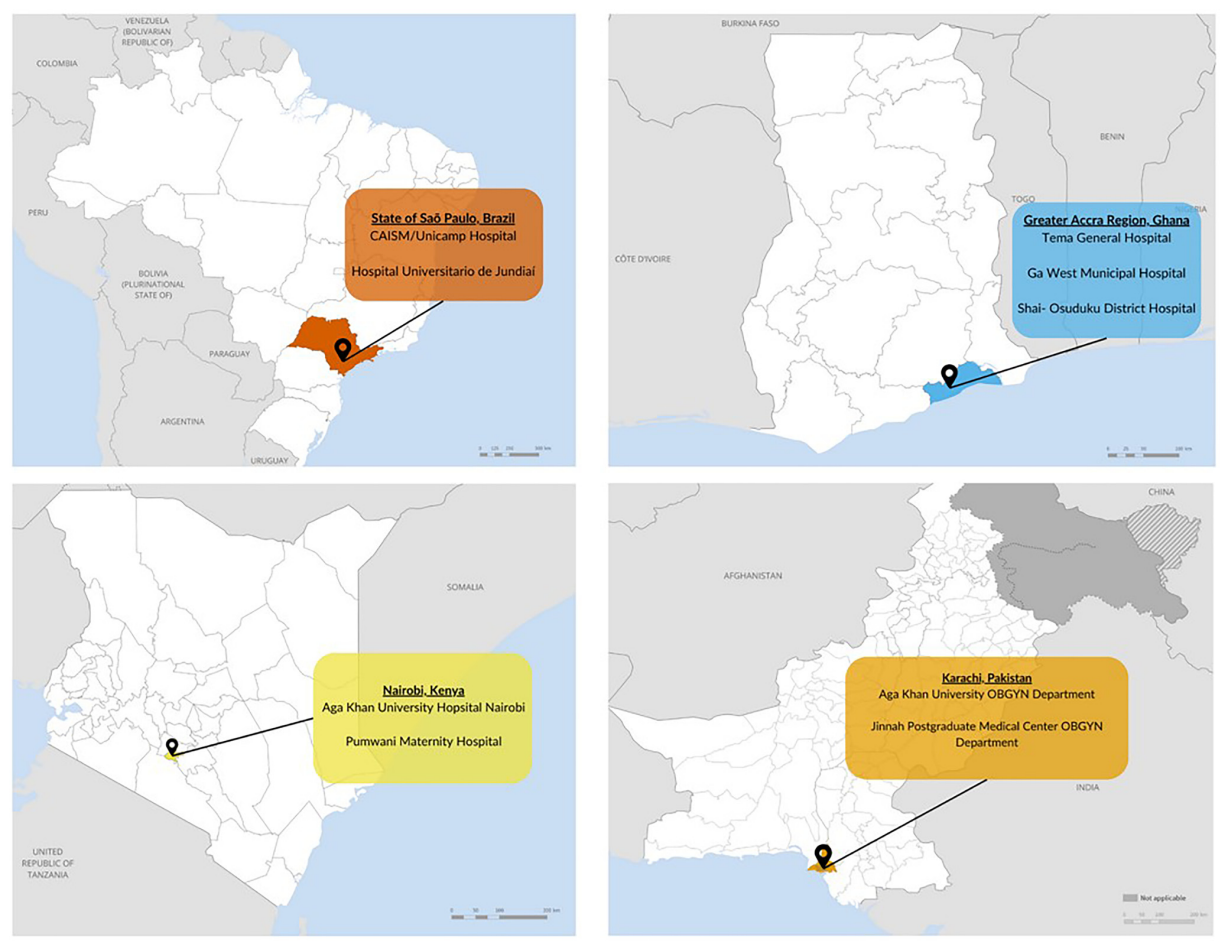
Study locations and clinic names in Brazil, Ghana, Kenya, and Pakistan.

Each of the four countries included in this study was chosen based on participation in a WHO-led multi-country cohort study of COVID-19 in pregnancy ( Broutet & Thorson, 2022) and various other factors when it was conceptualized in 2021, including geographic diversity, varying COVID-19 vaccine policies related to pregnant women, diversity of COVID-19 vaccine products available, phase of the COVID-19 epidemic, and country interest. Study locations within each country vary by clinic type, clinic level, and the population served. Within each country, sites were selected to ensure inclusion of perspectives from people living in urban and rural settings, from high and low socio-economic status, and/or seeking care at private or public clinics. ( Figure 2)

Brazil first introduced the COVID-19 vaccine in January 2021, and vaccination was only recommended for pregnant and lactating women with comorbidities who underwent a risk-benefit assessment by their physicians starting in March 2021 ( Covas *et al.*, 2023; Secretaria Extraordinária de Enfrentamento à COVID-19 Gabinete, 2021). Following the death of a pregnant Brazilian woman after receiving a dose of the AstraZeneca/Oxford (AZO) vaccine, the AZO vaccine was prohibited for use for pregnant women in May 2021 ( Covas *et al.*, 2023; Fonseca & Brito, 2021; Kobayashi *et al.*, 2022). Starting in September 2021, the Brazilian Ministry of Health (MoH) recommended Pfizer/BioNTech and Sinovac for all pregnant and lactating individuals, and the MoH continues to include pregnant and lactating women in their recommended COVID-19 vaccination schedules. 

Study sites in Brazil include two maternity hospitals in São Paulo, CAISM/Unicamp Hospital in Campinas, and Hospital Universitario de Jundiaí in Jundiaí, both public hospitals caring for pregnant women from urban and semirural areas and covered by the National Health Systems (SUS) and also private insurances.

Ghana was the first country to receive vaccines from the COVAX Facility in February 2021 ( UNICEF & World Health Organization, 2024). However, pregnant women were not included in the initial vaccine rollout, which focused on health workers and those with comorbidities, nor in the next two phases which expanded recommendations to include all adults over 18 years throughout 2021 ( The World Bank, 2021). Ghana only recommended COVID-19 vaccination for pregnant and lactating individuals after January 20, 2022, following updated guidance from WHO ( Berman Institute of Bioethics & Center for Immunization Research, 2022) . 

Study sites in Ghana are in the Greater Accra region. Three hospitals are included that represent regional, district, and secondary levels of care. These include Tema General Hospital, Ga West Municipal Hospital, and Shai-Osudoku District Hospital, each serving urban, mix of urban and rural, and rural populations, respectively.

Similarly to Ghana, Kenya introduced the COVID-19 vaccine with 1.02 million doses of COVAX-provided AZO vaccines in March 2021 ( World Health Organization, 2021a). Pregnant and lactating individuals were explicitly excluded from vaccination campaigns from February 2021 to January 2022, when the MoH revised its directives and recommended all COVID-19 vaccine types and brands for pregnant and lactating women ( Berman Institute of Bioethics & Center for Immunization Research, 2022; National Vaccine & Immunization Program, 2021). 

In Kenya, two antenatal and postnatal clinics in Nairobi were chosen: Aga Khan University Hospital Nairobi, a private referral hospital serving middle and higher socio-economic classes, and Pumwani Maternity Hospital, a public referral hospital that serves largely lower socio-economic status classes.

The first COVID-19 vaccines introduced to Pakistan were half a million doses of the Sinopharm vaccine donated by China in February 2021 ( Siddiqui *et al.*, 2021). Other COVID-19 vaccine brands, such as AZO were introduced in Pakistan via the COVAX Facility starting in May 2021 ( World Health Organization, 2021b). Unlike Brazil, Ghana, and Kenya, pregnant and lactating women were recommended for vaccination against COVID-19 from the beginning of the vaccine rollout, with the Special Minister to the Prime Minister on Health strongly urging all pregnant and lactating women to receive the vaccine following the deaths of two unvaccinated pregnant women from COVID-19 in August 2021 ( Berman Institute of Bioethics & Center for Immunization Research, 2022; Jajja, 2021).

Pakistan study sites include two hospitals in Karachi, a community private hospital, The Aga Khan Hospital for Women and Children, Kharadar, serving mostly people of lower and higher middle socio-economic community and Jinnah Postgraduate Medical Center, a tertiary care public hospital serving mostly low and lower-middle socio-economic community. 

### Sample size

For the qualitative component of the study, for each country, we aim to interview 25 pregnant and 25 postpartum women, for a total of 50 in-depth interviews per country, taking into consideration when data saturation might be reached. For those pregnant, we aim to interview approximately equal samples by trimester (1st, 2nd, and 3rd). For the quantitative component of the study, we aim to administer a survey to 400 pregnant women in each country to evaluate the proportion of participants with a given attitude and the comparison of attitude proportions by vaccination status. The sample size was determined with the following objectives and assumptions: 1) to evaluate the proportion of pregnant women with an attitude with 95% confidence intervals and 5% margin of error, assuming 50% of the population has the attitude (to provide maximum variability), and an unknown population size; 2) to compare two proportions with 95% confidence interval and 80% power, assuming 50% of the group 1 has the attitude and 40% of group 2 has the attitude. An unknown population size was assumed to facilitate evaluation across countries and the uncertainty in patient volume across facilities that sampling is occurring in.

In three of the four countries, Brazil, Kenya, and Pakistan, the goal is to sample approximately equal numbers of pregnant women across the three trimesters of pregnancy. In Ghana, due to cultural beliefs about seeking care in the 1st trimester, the target for the 1st trimester was decreased. In Brazil, Kenya, and Pakistan, an equal representation is being sought from each participating study clinic for both components of the study overall, but not necessarily for the trimester subgroup targets. All countries’ sample size and subgroup targets are listed in Table 1.

**Table 1.** Matrix of protocol components.

	SITES	SURVEY SAMPLE SIZE	IDI SAMPLE SIZE	SAMPLING STRATEGY	REMUNERATION
BRAZIL	2 maternity hospitals in São Paulo State	1st Tri:133 2nd Tri: 133 3rd Tri: 134	1st Tri: 8 2nd Tri: 9 3rd Tri: 8 Post: 25	Systematic	none
GHANA	3 maternity hospitals in Greater Accra Region	1st Tri: 40 2nd Tri: 180 3rd Tri: 180	1st tri: 8 2nd tri: 9 3rd tri: 8 Post: 25	Consecutive	70 GHS (~6 USD)
KENYA	2 referral maternity hospitals in Nairobi	1st Tri: 133 2nd Tri: 133 3rd Tri: 134	1st tri: 8 2nd tri: 9 3rd tri: 8 Post: 25	Consecutive	500 KES (~5 USD)
PAKISTAN	2 (1 maternity and 1 referral) hospitals in Karachi	1st Tri: 133 2nd Tri: 133 3rd Tri: 134	1st tri: 8 2nd tri: 9 3rd tri: 8 Post: 25	Consecutive	Meal box (value ~5 USD)

IDI: in-depth interview, Tri: pregnancy trimester, Post: post-partum, GHS: Ghana Cedi, USD: US Dollar, KES: Kenyan Shilling

### Recruitment

The recruitment strategy varies by country. Most sites are using a consecutive sampling method, approaching every eligible participant until they reach subgroup targets, alternating between the survey and the interview. In Brazil, both study sites are using systematic sampling, or sampling every *n*th person at the antenatal or postnatal clinics. The value of *n* is based on the patient volume of the clinic. At all three sites in Ghana, both sites in Kenya, and both sites in Pakistan, a consecutive sampling method of women in the clinic’s waiting area is used. In Brazil, participants can join both components of the study (quantitative and qualitative). In Ghana, Kenya, and Pakistan, participants can join only one component of the study. Three of the countries, Ghana, Kenya, and Pakistan, are providing some type of remuneration to reimburse participants’ travel cost or thank you gift to participants. Brazil is not providing any renumeration due to ethical constraints. In Brazil, reimbursement is only accepted if extra costs are incurred by participating in the study, which do not apply to this study.

Recruitment starts with study staff approaching potentially eligible persons in the waiting or reception area of the health care facility. The study staff reads a study recruitment script to the potential participant in a semi-private area. At the end of the recruitment script is an eligibility screen. Eligibility in this study is broad and include five criteria: 1) pregnant or up to six weeks postpartum (interview only), 2) study interest, 3) age of 18 or older (or an emancipated minor in Brazil only), 4) fluent in the local language (or English if applicable), and 5) knowledge of the COVID-19 vaccine. After passing the eligibility questions, the script also asks for their trimester of pregnancy and COVID-19 vaccination status. The trimester of pregnancy question is used to fill the trimester quotas defined in the sample size targets. While there are no sample size targets for vaccinated and unvaccinated within any of the countries, the study aspires to obtain a representation of vaccinated and unvaccinated participants across all four countries. If eligibility is met and the sub-group is needed, the study staff member invites the participant to join. If the prospective participant agrees, informed consent occurs in a private location followed by data collection. Study staff ensure that the participant’s clinic appointment is not missed due to study participation and pauses any study activities if the participant is called to see a provider. Study participation only restarts after the visit is complete.

### Data collection

Data collection instruments, surveys and interview guides, were developed through an iterative process that started with a review of the literature, including a review of relevant instruments ( Alagarsamy *et al.*, 2022; Betsch *et al.*, 2018; Bronfenbrenner, 1979; Larson *et al.*, 2015; Rosenstock *et al.*, 1988). They were then reviewed by country teams, and pre-tested in each country among data collectors before finalization. Each of the four country teams was able to amend the questionnaire and interview guides to better align to local contexts while efforts were made to ensure sufficient data would be available for pooled, cross-country analyses.

The questionnaire was developed to identify attitudinal, behavioral, and social correlates of vaccine uptake and we sought to use validated items or adapt validated items ( Alagarsamy *et al.*, 2022; Betsch *et al.*, 2018; Bronfenbrenner, 1979; Larson *et al.*, 2015; Rosenstock *et al.*, 1988). The questionnaire contains questions on socio-demographics, attitudes toward COVID-19 vaccines, COVID-19 vaccine knowledge and information sources, COVID-19 vaccine behaviors and intentions, and general attitudes towards vaccination in pregnancy, including receipt of other maternal vaccines that might become available in the future.

The in-depth interview guide includes topics on the decision-making process related to COVID-19 vaccines, including risk perception, self-efficacy, vaccine intentions, and social norms, etc. Two interview guides were developed, one for pregnant and one for postpartum participants.

Questions related to the following constructs are included: influences of decision-making, self-efficacy, norms, risk perception, knowledge of disease, knowledge of vaccines, information sources, and vaccine hesitancy.

In Brazil and Pakistan, questionnaires and interviews are done by two separate data collection teams. In Ghana and Kenya, both components of the study are done by one data collection team. All countries are digitally audio recording the qualitative interviews; Pakistan is also including a note-taker in each of the interviews. Brazil and Kenya are using paper-based data collection and double data entry for all questionnaires. Ghana and Pakistan are using tablet-based data collection using either the REDCap Mobile Application or REDCap’s web-based data entry interface. All study data, including in-depth interview audio files, are managed and stored using REDCap electronic data capture tools hosted at JHU ( Harris *et al.*, 2009; Harris *et al.*, 2019). REDCap (Research Electronic Data Capture, Nashville, TN, USA: https://projectredcap.org) is a secure, web-based software platform designed to support data capture for research studies that is available to non-profit groups who join the consortium. Alternatives that are also free for non-profit groups include Kobo Toolbox (Cambridge, MA. USA: https://www.kobotoolbox.org) and a self-managed version of Open Data Kit (Seattle, WA, USA: https://getodk.org). Data collection is done in Brazilian Portuguese in Brazil; Ga, Twi, or English in Ghana; Kiswahili or English in Kenya; and Urdu or English in Pakistan. Both components of the study (questionnaires and interviews) are estimated to take 30-60 minutes to complete, inclusive of the time needed for the consent process.

### Data analysis and statistical plan

In Brazil, Kenya, and Pakistan, audio files from qualitative interviews are transcribed in the language they were completed in and then translated to English. In Ghana, transcripts are typed directly into English given the colloquial nature of Ga and Twi languages. Any notes that are taken during the interview are incorporated during transcription. All transcriptions and translations undergo review by an independent study team member as part of standard practice. Questionnaire data are reviewed and cleaned following a standardized data cleaning procedure. No personally identifying information (PII) is captured during the questionnaire and while no PII is intentionally captured during interviews, an anonymization procedure is being followed during transcription to ensure no PII is included in the final transcripts.

For the qualitative aim of the study, a grounded theory approach is followed for data processing and analyses. Each country undergoes an independent and iterative open coding process with representatives from the country team, JHU, and WHO. A minimum of two open coding sessions are conducted to develop and refine a codebook for analysis. Participants for each open coding session review the same random selection of transcripts and through an inductive coding approach, a final codebook is generated. After all countries complete their codebooks, a final code structure and thematic categories will be selected; these will be applied to each transcript in the final coding process. All transcript coding is done with ATLAS.ti ( Smit, 2002). An alternative open access qualitative coding platform is Taguette, https://www.taguette.org ( Rampin & Rampin, 2021).

*A-priori* analyses for pooled cross-country qualitative data fall into three topic areas, outlined in Table 2. For the quantitative aim of the study, there are four main topic areas for planned analyses and the questionnaire was structured around these four themes ( Table 2). The primary aims of this study are descriptive and are covered by the planned analyses of both the qualitative and quantitative components of the study. Country specific analyses will be defined and led by each of the country teams.

**Table 2.** Planned analyses for pooled, cross-country data.

ANALYSIS TOPIC AREA	ANALYSIS TYPE/ FRAMEWORK	STUDY COMPONENT
COVID-19 vaccine knowledge and information sources	Descriptive	Quantitative
COVID-19 vaccine intentions and behaviors during pregnancy	Descriptive	Quantitative
Attitudes toward future maternal vaccines	Descriptive	Quantitative
Attitudes toward COVID-19 vaccines	Descriptive	Quantitative
COVID-19 vaccination awareness and behaviors during pregnancy	Grounded Theory	Qualitative
COVID-19 awareness, risk perception, and mitigation	Grounded Theory	Qualitative
Vaccination experiences generally and specifically in pregnancy	Grounded Theory	Qualitative

### Ethical review

Ethical review and approval for the 4-country study was sought from the Johns Hopkins Bloomberg School of Public Health Institutional Review Board (Ref. IRB00020864, approved 2023-07-06; Ref. IRB00020850, approved 2023-09-12; Ref. IRB00020861, approved 2023-09-27; Ref. IRB00020866, approved 2024-02-01), and the World Health Organization’s Research Ethics Review Committee (Ghana: Ref. CERC.0193A, approved 2023-06-05; Kenya: Ref. CERC.0193B, approved 2023-06-19; Pakistan: Ref. CERC.0193C, approved 2023-09-19) or the Pan American Health Organization (Brazil: Ref. PAHOERC.0633.01, approved 2023-03-24),. Each country protocol also underwent scientific review through the WHO/HRP Research review research panel (Switzerland). Individual country teams sought and obtained approvals for each country-level research plan with the following entities: Committee of Research Ethics from the University of Campinas (Brazil: Ref. 63968222.1.1001.5404, approved 2023-04-10), Jundiaí University Institutional Review Board (Brazil: Ref. CAAE 63968222.1.2001.5412, approved 2023-07-07), Ghana Health Service Ethics Review Committee (Ghana: Ref. 028/03/23, approved 2023-05-23), The Aga Khan University’s Institutional Scientific and Ethics Committee (Kenya: Ref. 2023/ISERC-17, approved 2023-06-19), Pumwani maternity hospital ethics review committee (Kenya: Ref. PMH/CEO/76/0785/2023, approved: 2023-12-13), the National Council for Science Technology and Innovation (Kenya: Ref. NACOSTI/P/23/29152, approved 2023-09-27), Nairobi County Research and Development Committee (Kenya: Ref. NCC/CS/RPD/84/2023, approved 2023-11-27), National Bioethics Committee (Pakistan: Ref. No.4-87/NBCR-1029/23, approved 2024-01-03), and The Aga Khan University Institutional Ethics Review Committee (Pakistan: Ref. 2023-8633-25854, approved 2023-07-27), and the Institutional Review Board at Jinnah Postgraduate Medical Center (Pakistan: Ref. F.2-81/2023-GENL/182/JPMC, approved 2023-12-14).

All study staff were trained in human subjects’ research ethics as well as qualitative and/or quantitative data collection during a three-day country-specific training session. Qualitative training included interviewing techniques to reduce bias, transcription, and translation. Participants in all four countries were recruited in semi-private areas of the clinic and underwent an informed consent process with trained study team members in private areas. All four countries used written informed consent, using alternatives for illiterate participants as allowed by each country. A transcription standardized operating procedure was developed and will be used by all country teams to ensure that all personally identifying information is removed from final transcripts. Standard data cleaning procedures will also be used by all countries.

### Dissemination plans

The results of the research will be submitted to peer-reviewed publications in specialized journals and to scientific dissemination meetings and congresses.

In Brazil, at the national and regional level, dissemination will be done through conferences and reports to policy makers to inform strategies and gaps related to the topic. The investigators involved in the study in Brazil are part of National and Regional policy-making committees in maternal and perinatal health and they will work with local partners and stakeholders to develop local dissemination plans. In Ghana, prior to publication, preliminary findings will be disseminated to study facilities. The data and findings from the study will also be disseminated to the Ghana Health Service and other key stakeholders to inform context-specific guidelines for vaccine decision-making and uptake among pregnant and postpartum women in Ghana.

In Kenya, the results from this project will be used for advocacy with health managers and policy makers focusing on the best demand generation and communication strategies to improve the uptake of COVID-19 vaccines for pregnant women. The research team will disseminate the findings in an organized forum comprising different cadres of ministry of health personnel at policy and practice level as well as other relevant stakeholders involved with health care service provision in Kenya. Furthermore, the research team will develop policy briefs for the policy makers and peer reviewed publications in international journals for wider dissemination. In Pakistan, the research findings will be shared with relevant stakeholders, including policymakers and leading obstetricians, through peer-reviewed journals, provincial/national-level seminars, and the meetings of the Society of Obstetricians and Gynecologists of Pakistan (SOGP). The aim is to maximize the visibility and impact of the research findings and contribute to informed decision-making and improved healthcare practices in Pakistan.

## Conclusion/discussion

With COVID-19 vaccine uptake during pregnancy lagging behind its recommendations for use, the lessons learned from this study can help inform future COVID-19 vaccine delivery and communications strategies. In addition, as several new maternal vaccines are in the late stages of development or the earliest stages of implementation, this study can also help to inform future vaccine introductions. These findings will also be useful for global policy makers to understand how important factors related to maternal vaccine uptake vary by location, as well as contextual factors that should be considered in program implementation. For policy makers at the national and local level, these data can inform strategies to improve maternal vaccination acceptance and coverage and encourage execution of similar studies in other settings to learn about specific local contexts.

## Study status

Data collection was completed in all countries as of 25 May 2024. Data processing, cleaning, and analysis are underway. No data has been published from this study at the time of writing. Results will be presented in subsequent publications. 

## Ethics and consent

Ethical review and approval for the 4-country study was sought from the Johns Hopkins Bloomberg School of Public Health Institutional Review Board (Ref. IRB00020864, approved 2023-07-06; Ref. IRB00020850, approved 2023-09-12; Ref. IRB00020861, approved 2023-09-27; Ref. IRB00020866, approved 2024-02-01), and the World Health Organization’s Research Ethics Review Committee (Ghana: Ref. CERC.0193A, approved 2023-06-05; Kenya: Ref. CERC.0193B, approved 2023-06-19; Pakistan: Ref. CERC.0193C, approved 2023-09-19) or the Pan American Health Organization (Brazil: Ref. PAHOERC.0633.01, approved 2023-03-24),. Each country protocol also underwent scientific review through the WHO/HRP Research review research panel (Switzerland).Individual country teams sought and obtained approvals for each country-level research plan with the following entities: Committee of Research Ethics from the University of Campinas (Brazil: Ref. 63968222.1.1001.5404, approved 2023-04-10), Jundiaí University Institutional Review Board (Brazil: Ref. CAAE 63968222.1.2001.5412, approved 2023-07-07), Ghana Health Service Ethics Review Committee (Ghana: Ref. 028/03/23, approved 2023-05-23), The Aga Khan University’s Institutional Scientific and Ethics Committee (Kenya: Ref. 2023/ISERC-17, approved 2023-06-19), Pumwani maternity hospital ethics review committee (Kenya: Ref. PMH/CEO/76/0785/2023, approved: 2023-12-13), the National Council for Science Technology and Innovation (Kenya: Ref. NACOSTI/P/23/29152, approved 2023-09-27), Nairobi County Research and Development Committee (Kenya: Ref. NCC/CS/RPD/84/2023, approved 2023-11-27), National Bioethics Committee (Pakistan: Ref. No.4-87/NBCR-1029/23, approved 2024-01-03), and The Aga Khan University Institutional Ethics Review Committee (Pakistan: Ref. 2023-8633-25854, approved 2023-07-27), and the Institutional Review Board at Jinnah Postgraduate Medical Center (Pakistan: Ref. F.2-81/2023-GENL/182/JPMC, approved 2023-12-14).

All four countries used written informed consent, using alternatives for illiterate participants as allowed by each country

## Data Availability

No data are associated with this article. Data collected during this study will be made available when results are published as allowed by the data sharing policies of the individual institutions that led data collection in each of the four participating countries. Open Science Framework: Exploring Knowledge, Attitudes, and Practices Related to Vaccine Decision-Making among Pregnant People, DOI 10.17605/OSF.IO/G3YD2 ( Schue, 2024). This project contains the following extended data: Consent Form Interview: Written consent form for interview participants. Consent Form Survey: Written consent form for survey participants. Master Post-Pregnancy IDI Guide: Semi-structured interview guide for post-pregnant women Master Pregnancy IDI Guide: Semi-structured interview guide for pregnant women Survey Master: Survey instrument for pregnant women License: *CC-By Attribution 4.0 International*
