# Supplementary material for: Vaccine decision-making among pregnant women: a protocol for a cross-sectional mixed-method study in Brazil, Ghana, Kenya and Pakistan
Source: Gates Open Res. 2024 Oct 21;8:94. Originally published 2024 Aug 29. [Version 2] doi: 10.12688/gatesopenres.16280.2 (PMC11489405; doi:10.12688/gatesopenres.16280.2)
Supplement: Peer review history

# Reviewer report: Reviewer response for version 2

**DOI:** 10.21956/gatesopenres.17694.r39219

**Published:** 2025-01-20

**Authors:** David A Schwartz

**Affiliations:** 1 Perinatal Pathology Consulting, Atlanta, USA

This is an original proposal for utilizing a cross-sectional mixed methods approach to determine vaccine decision-making among pregnant and postpartum women in 4 countries. As would be expected by the expertise of the investigators, it is a well-organized and well-planned study that uses a variety of quantitative and qualitative methods to determine the factors that are important to pregnant and postpartum women when deciding on vaccine uptake.

 This is a crucially important study with the continuing waves of vaccine-preventable infections that have been occurring throughout the world - including those from emerging and reemerging viral agents such as SARS-CoV-2 and mpox virus.

 There is no need for me to repeat the minor critiques and suggestions that have been put forward by the other reviewers of this useful investigation. 

 I would suggest that the authors consider adding a sentence or two to the Introduction that addresses a very important issue that arose during the initial phases of the COVID-19 pandemic. Prior to the development of the Omicron strains, stillbirth and neonatal death was an important problem among pregnant women with COVID-19. Based on a number of multinational placental pathology/clinical outcomes studies, this was found to largely be the result of placental insufficiency caused by massive placental destruction by the virus, causing SARS-CoV-2 placentitis. It was found that essentially all cases of this occurred in nonvaccinated mothers, and that COVID-19 vaccination drastically reduced the incidence of this placental lesion and stillbirth (consider citing reference below from the *Amer J Ob Gyn*). Thus, maternal vaccination can be lifesaving for the fetus while still in the womb.

Is the study design appropriate for the research question?

Yes

Is the rationale for, and objectives of, the study clearly described?

Yes

Are sufficient details of the methods provided to allow replication by others?

Yes

Are the datasets clearly presented in a useable and accessible format?

Yes

Reviewer Expertise:

Obstetric, placental and perinatal pathology; usage and effects of vaccination for emerging infections on pregnant women, the fetus and placenta; maternal and fetal mortality due to emerging infections; pathophysiology of stillbirth and neonatal death; medical epidemiology

I confirm that I have read this submission and believe that I have an appropriate level of expertise to confirm that it is of an acceptable scientific standard.

## References

[1] : SARS-CoV-2 placentitis, stillbirth, and maternal COVID-19 vaccination: clinical-pathologic correlations. *Am J Obstet Gynecol* .2023;228(3) : 10.1016/j.ajog.2022.10.001 261-269 10.1016/j.ajog.2022.10.001 36243041 PMC9554221

# Reviewer report: Reviewer response for version 2

**DOI:** 10.21956/gatesopenres.17694.r39136

**Published:** 2024-12-31

**Authors:** Waldemar A. Carlo

**Affiliations:** 1 University of Alabama at Birmingham, Birmingham, AL, USA

General comments

 This cross-sectional multi-country study among pregnant and postpartum women is designed to understand their decision-making process for vaccine intake including their related knowledge, attitudes, and behaviors. This is a well-designed study as it includes participants from diverse settings including countries with geographic diversity, varying COVID-19 vaccine policies related to pregnant women, diversity of COVID-19 vaccine products available, and phase of the COVID-19 epidemic. Participants within each country vary to ensure inclusion of perspectives from people living in urban and rural settings, from high and low socioeconomic status, and/or seeking care at private or public clinics.

 This study addresses the important subject of vaccine-making process by pregnant and postpartum women. A limitation is that the COVID vaccine may have changed attitudes of participants because of many circumstances that were particular to this disease that may not be applicable to diseases covered by other vaccines such as tetanus, etc., that are more established as the specific results may have limited generalizability to other vaccines.  

 I assessed all aspects of the manuscript.

 Introduction

 The pertinent most recent literature is reviewed, and five clear objectives are defined.

 Methods

 The study design and locations are sound. The sample size is well justified.

 Data collection included quantitative data obtained with a survey questionnaire designed to identify important attitudes and beliefs associated with vaccination and qualitative data obtained with in-depth interviews designed to obtain data on beliefs and attitudes.

 Conclusions/Discussion

 It may be best to address possible limited generalizability of the COVID-19 vaccine results to other vaccines because of particular issues related to the COVID vaccine and pandemic including the new type of vaccine, the urgency in which the vaccine was developed, the limited testing before vaccination was implemented, and the political involvement in many countries that overshadowed the best public health strategies and practices.

Is the study design appropriate for the research question?

Yes

Is the rationale for, and objectives of, the study clearly described?

Yes

Are sufficient details of the methods provided to allow replication by others?

Yes

Are the datasets clearly presented in a useable and accessible format?

Yes

Reviewer Expertise:

Perinatal and postnatal, pregnancy, pregnancy and birth risks, neonatology, maternal-fetal medicine, prematurity, morbidity and mortality, research in these areas as well as neonatal clinical practice, COVID-19 vaccine studies for neonates, and pregnant/postpartum women.

I confirm that I have read this submission and believe that I have an appropriate level of expertise to confirm that it is of an acceptable scientific standard.

# Reviewer report: Reviewer response for version 2

**DOI:** 10.21956/gatesopenres.17694.r38297

**Published:** 2024-11-12

**Authors:** Edina Amponsah-Dacosta, Imen Ayouni Ep Labidi

**Affiliations:** 1 Public Health, University of Cape Town, Western Cape, South Africa; 2 Pediatrics and Child Health, University of Cape Town Faculty of Health Sciences, Observatory, Western Cape, South Africa

There are pending aspects that the authors have not adequately responded to. For example, given that this is a protocol and to enhance transparency and reproducibility, it is important that a comprehensive data analysis is provided giving guidance on the intended analytical approach. A limitations section will provide clarity on aspects of the methodological approach that the authors and researchers have carefully and objectively considered including any anticipated risks.

Is the study design appropriate for the research question?

Yes

Is the rationale for, and objectives of, the study clearly described?

Yes

Are sufficient details of the methods provided to allow replication by others?

Partly

Are the datasets clearly presented in a useable and accessible format?

Yes

Reviewer Expertise:

Medical Virology, Vaccinology, and Health Policy and Systems Research

We confirm that we have read this submission and believe that we have an appropriate level of expertise to confirm that it is of an acceptable scientific standard, however we have significant reservations, as outlined above.

# Reviewer report: Reviewer response for version 2

**DOI:** 10.21956/gatesopenres.17694.r38299

**Published:** 2024-10-25

**Authors:** Thiago Cerqueira-Silva

**Affiliations:** 1 Faculty of Epidemiology and Population Health, London School of Hygiene & Tropical Medicine, London, UK

The authors did not reply to the points previously raised.

 Especially about the equity of the research.

Is the study design appropriate for the research question?

Yes

Is the rationale for, and objectives of, the study clearly described?

Yes

Are sufficient details of the methods provided to allow replication by others?

Partly

Are the datasets clearly presented in a useable and accessible format?

Not applicable

Reviewer Expertise:

Infectious diseases' epidemiology, biostatistics, COVID-19, vaccines

I confirm that I have read this submission and believe that I have an appropriate level of expertise to state that I do not consider it to be of an acceptable scientific standard, for reasons outlined above.

# Response

**Published:** 2024-10-25

**Authors:** Jessica Schue

**Affiliations:** International Health, Johns Hopkins University Department of International Health, Baltimore, Maryland, USA

Thank you for this review. Unfortunately, Version 2 was published before you completed your initial review, as we were instructed by the editors to do so. We will consult with the editors on how best to proceed. We may await the other two reviewers to review the current version before addressing your original comments to ensure that everyone's comments are included in the next revision.

# Reviewer report: Reviewer response for version 1

**DOI:** 10.21956/gatesopenres.17645.r38016

**Published:** 2024-10-18

**Authors:** Thiago Cerqueira-Silva

**Affiliations:** 1 Faculty of Epidemiology and Population Health, London School of Hygiene & Tropical Medicine, London, UK

This protocol outlines a multi-country, cross-sectional mixed-method study that aims to explore vaccine decision-making among pregnant and postpartum women using qualitative and quantitative methods. The findings should be useful for designing vaccination strategies and policies for pregnant women.

 One of my main concerns is the equity aspect of this research. The study is conducted in four countries—Brazil, Ghana, Kenya, and Pakistan—yet none of the first or last four authors of the protocol are from any of these countries. If this pattern continues in subsequent manuscripts from the research, it may fall under parachute or helicopter research. The authors should address how they intend to ensure equitable partnerships. (Morton B, et al., 2022 [Ref 1])

 My second major point relates to the study's timeline. The protocol states that data collection is already complete, so it is unclear the rationale for publishing the study protocol after data collection and if any previous version of the protocol was published elsewhere (OSF or related).

 In addition, I have a few minor points regarding the protocol:

 1. The information on COVID-19 vaccination in Brazil is inaccurate. The Ministry of Health resumed vaccination for pregnant women in July 2021, not September (as detailed here: https://www.gov.br/pt-br/noticias/saude-e-vigilancia-sanitaria/2021/07/governo-recomenda-vacinacao-contra-covid-19-em-gestantes-e-puerperas-sem-comorbidades). It should also be clarified that the death mentioned was related to thrombosis following an AstraZeneca vaccine.

 2. In the recruitment section, there is no explanation for the differences in systematic versus consecutive recruitment approaches between countries.

 3. Eligibility Criterion 5 is unclear in defining what is meant by "knowledge of the COVID-19 vaccine."

 4. In the Data Collection section, the type of “pre-test” conducted by the teams is not specified. The final questionnaire should be included as supplementary material to allow an assessment of the protocol's feasibility.

 Finally, the dissemination plan does not mention any outreach to the general public.

Is the study design appropriate for the research question?

Yes

Is the rationale for, and objectives of, the study clearly described?

Yes

Are sufficient details of the methods provided to allow replication by others?

Partly

Are the datasets clearly presented in a useable and accessible format?

Not applicable

Reviewer Expertise:

Infectious diseases' epidemiology, biostatistics, COVID-19, vaccines

I confirm that I have read this submission and believe that I have an appropriate level of expertise to confirm that it is of an acceptable scientific standard, however I have significant reservations, as outlined above.

## References

[1] : Consensus statement on measures to promote equitable authorship in the publication of research from international partnerships. *Anaesthesia* .2022;77(3) : 10.1111/anae.15597 264-276 10.1111/anae.15597 PMC929323734647323

# Reviewer report: Reviewer response for version 1

**DOI:** 10.21956/gatesopenres.17645.r38012

**Published:** 2024-09-25

**Authors:** Lucía Abascal Miguel

**Affiliations:** 1 Institute for Global Health Sciences, University of California, California, USA

This protocol outlines a multi-country, cross-sectional mixed-method study that aims to explore vaccine decision-making among pregnant and postpartum women in Brazil, Ghana, Kenya, and Pakistan, focusing on the COVID-19 vaccine. The study employs both quantitative surveys and qualitative interviews to gather insights on attitudes, behaviors, and social correlates influencing vaccine uptake during and after pregnancy. The research also aims to understand how risk perception, self-efficacy, and social norms contribute to decision-making processes regarding maternal immunization. The findings from this study are expected to inform national and international vaccination strategies and policies for pregnant women.

 Regarding methods: 

 - While the study provides a clear description of the mixed-methods approach, including both qualitative and quantitative components, there are some areas that could benefit from more detailed clarification. For example, the selection criteria for the study sites within each country, particularly how diversity in socio-economic or urban/rural status will be  ensured, could be elaborated further. Additionally, more details on how participant confidentiality will be maintained during the consent process would strengthen the methods section.

 - **Datasets presented** is not applicable since the study is still in the protocol stage, meaning the datasets have not yet been presented.

 - Other recommendations: 

**Survey Design**: The article mentions that the questionnaire has been adapted to local contexts. It might be beneficial for the authors to include more details on how these adaptations vary across countries and what cultural factors were considered.

Is the study design appropriate for the research question?

Yes

Is the rationale for, and objectives of, the study clearly described?

Yes

Are sufficient details of the methods provided to allow replication by others?

Partly

Are the datasets clearly presented in a useable and accessible format?

Not applicable

Reviewer Expertise:

Vaccine uptake, vaccine hesitancy, COVID-19 vaccine access, global health

I confirm that I have read this submission and believe that I have an appropriate level of expertise to confirm that it is of an acceptable scientific standard.

# Response

**Published:** 2024-10-17

**Authors:** Jessica Schue

**Affiliations:** International Health, Johns Hopkins University Department of International Health, Baltimore, Maryland, USA

Thank you for reviewing this article. We appreciate your suggestions and have addressed the concerns you had in the initial review. 

- **Reviewer 2:** While the study provides a clear description of the mixed-methods approach, including both qualitative and quantitative components, there are some areas that could benefit from more detailed clarification. For example, the selection criteria for the study sites within each country, particularly how diversity in socio-economic or urban/rural status will be ensured, could be elaborated further. Additionally, more details on how participant confidentiality will be maintained during the consent process would strengthen the methods section.
  - Thank you for this comment. We clarified that facilities were also a part of the parent WHO cohort study, and within that limited set, the sites were chosen to maximize the diversity of participants. We also added in more information on how confidentiality was kept.
- **Reviewer 2: Survey Design**: The article mentions that the questionnaire has been adapted to local contexts. It might be beneficial for the authors to include more details on how these adaptations vary across countries and what cultural factors were considered.
  - Thank you for flagging this. There were only minor changes to both instruments, and none of them impacted the results that will be presented. In most cases, the changes were done to a question’s structure to improve its comprehension after translation. The other changes were related to some of the socio-demographic variables, Kenya’s questionnaire did not include Ethnicity, Brazil’s questionnaire included an age group of <18 and emancipated, and Pakistan’s questionnaire excluded the gender and marital status questions.  

# Reviewer report: Reviewer response for version 1

**DOI:** 10.21956/gatesopenres.17645.r37873

**Published:** 2024-09-20

**Authors:** Edina Amponsah-Dacosta, Imen Ayouni Ep Labidi

**Affiliations:** 1 Public Health, University of Cape Town, Western Cape, South Africa; 2 Pediatrics and Child Health, University of Cape Town Faculty of Health Sciences, Observatory, Western Cape, South Africa

**Reviewer’s Comments**

 Thank you for the opportunity to review this manuscript which reports on a study protocol for a mixed-methods research project investigating vaccine decision-making among pregnant and postpartum women in Brazil, Ghana, Kenya, and Pakistan, with a focus on COVID-19 vaccines. The study aims to understand the attitudes, behaviours, and social factors influencing vaccine uptake during pregnancy. The multi-country design allows for cross-cultural comparisons and broader generalizability, providing insights into vaccine decision-making across diverse contexts. The research focus is timely and important given the global health challenges of COVID-19 and the under-development of maternal immunization programs in low- and middle- income countries. Anticipated findings from this study are expected to guide strategies for improving maternal vaccine uptake, particularly in low- and middle-income countries.

 I have the following comments which need addressing:

- From the methods section, it is unclear if data collection tools have or will be piloted. As such, it is not possible to critically assess the feasibility and validity of data collection tools and processes. A clear indication of the details of the pilot phase should be appropriately described as part of the methods section.
- As part of the study design, the authors should include a justification for the mixed methods approach, highlighting why this is best suited to address the core objectives of the study.
- Page 5: “Following the death of a pregnant Brazilian woman…” For clarity, it is crucial that the authors comment on the direct / indirect link between the vaccine administered and the case of death drawing from the studies cited. As it stands it is unclear to the reader whether investigations into this SAEFI was directly linked to the vaccine/vaccination.
- Page 5: “…pregnant and lactating women were recommended for vaccination against COVID-19…” Kindly rephrase this to indicate that vaccination was recommended for pregnant and lactating women and not the women for vaccination.
- Page 5: “…following the deaths of two unvaccinated pregnant women from COVID-19…” Suggestion, rephrase to "...from complications associated with COVID-19...”
- Page 6: “…to evaluate the proportion of participants with a given attitude and…” RE: “…given attitude…”, kindly qualify this by providing examples of the attitudes of interest in parenthesis.
- Page 7: “Eligibility in this study is broad and include five criteria…” Replace “include” with “includes”.
- Page 7: “Eligibility in this study is broad and include five criteria… 2) study interest…” It is unclear to me why “study interest” will be an eligibility criterion. What do the authors imply by "study interest"? Wouldn't the study automatically include only those participants who indicate a willingness to participate and provide informed consent? How would pregnant women be screened for study interest?
- Page 7: “Eligibility in this study is broad and include five criteria… 5) knowledge of the COVID-19 vaccine.” Kindly expand on why women with no knowledge of the COVID-19 vaccine will be excluded from the study.
- Page 7: “…the script also asks for … COVID-19 vaccination status.” The authors should clarify if this will be based on patient recall only or corroborated by valid vaccination records. Where limitations arise with regards to the data source, do ensure that this is appropriately addressed in a study limitations section.
- Page 8: “Questions related to the following constructs are included…disease, knowledge of vaccines…” Given that women who demonstrate no or low knowledge of COVID-19 vaccines would be ineligible to participate, how would this component feature in the study?
- Page 8: “All countries are digitally audio recording… Brazil and Kenya are using paper-based data collection…” The authors should comment on how the diverse data collection formats could influence the accuracy and quality of data collected and the measures to be taken for consolidation of data across participating countries during the analysis stage. In addition, the authors should address how they will treat missing data.
- Page 8: “Both components of the study (questionnaires and interviews) …” RE: Questionnaires, the authors should clarify if there will be back translation.
- Page 8: “For the qualitative aim of the study, a grounded theory approach…” Kindly provide a justification for this approach and why it is best suited for this study.
- The data analysis approach for the quantitative arm of the study is insufficiently addressed. Kindly provide a detailed analysis plan, highlighting any variations across countries / settings.
- As part of the “Dissemination plans”, the authors should address how study findings will be shared with participants and immediate affected communities (i.e., what arrangements have been made for community engagement and feedback?).
- The authors should consolidate the "Ethical review" and the “Ethics and consent” sections to avoid repetition.
- Page 10: “All four countries used written informed consent, using alternatives for illiterate participants…” Kindly specify the alternative approaches adopted for the consenting process for participants who were unable to read or write.
- The authors should include a study limitations section, comprehensively addressing all limitations associated with the design, methodological tools and approaches, and execution of this study.

Is the study design appropriate for the research question?

Yes

Is the rationale for, and objectives of, the study clearly described?

Yes

Are sufficient details of the methods provided to allow replication by others?

Partly

Are the datasets clearly presented in a useable and accessible format?

Yes

Reviewer Expertise:

Medical Virology, Vaccinology, and Health Policy and Systems Research

We confirm that we have read this submission and believe that we have an appropriate level of expertise to confirm that it is of an acceptable scientific standard, however we have significant reservations, as outlined above.

# Response

**Published:** 2024-10-15

**Authors:** Jessica Schue

**Affiliations:** International Health, Johns Hopkins University Department of International Health, Baltimore, Maryland, USA

Response to Reviewer 1 concerns, original comments are included for readability: 

- From the methods section, it is unclear if data collection tools have or will be piloted. As such, it is not possible to critically assess the feasibility and validity of data collection tools and processes. A clear indication of the details of the pilot phase should be appropriately described as part of the methods section.
  - Thank you for flagging this. Under “Data Collection” we state that tools were pre-tested by each country team prior to finalization.
- As part of the study design, the authors should include a justification for the mixed methods approach, highlighting why this is best suited to address the core objectives of the study.
  - Thank you, this clarification has been added to the study design section.
- Page 5: “Following the death of a pregnant Brazilian woman…” For clarity, it is crucial that the authors comment on the direct / indirect link between the vaccine administered and the case of death drawing from the studies cited. As it stands it is unclear to the reader whether investigations into this SAEFI was directly linked to the vaccine/vaccination.
  - We have clarified in the text that the decision to prohibit the AZO vaccine was made *before* a causal association was established. Whether causal or not, the purpose of pointing this out is to provide relevant background on the local circumstances that may influence vaccine attitudes. 
- Page 5: “…pregnant and lactating women were recommended for vaccination against COVID-19…” Kindly rephrase this to indicate that vaccination was recommended for pregnant and lactating women and not the women for vaccination.
  - We have rephrased this.
- Page 5: “…following the deaths of two unvaccinated pregnant women from COVID-19…” Suggestion, rephrase to "...from complications associated with COVID-19...”
  - Thank you for the suggestion, we have rephrased to indicate the deaths were due to complications from COVID-19.
- Page 6: “…to evaluate the proportion of participants with a given attitude and…” RE: “…given attitude…”, kindly qualify this by providing examples of the attitudes of interest in parenthesis.
  - We have included an example and referenced the supplementary material.
- Page 7: “Eligibility in this study is broad and include five criteria…” Replace “include” with “includes”.
  - Thank you, this has been updated.
- Page 7: “Eligibility in this study is broad and include five criteria… 2) study interest…” It is unclear to me why “study interest” will be an eligibility criterion. What do the authors imply by "study interest"? Wouldn't the study automatically include only those participants who indicate a willingness to participate and provide informed consent? How would pregnant women be screened for study interest?
  - Participants were given brief information about the study as part of recruitment, after learning about what the study entails, they were asked if they are interested in the study. 
- Page 7: “Eligibility in this study is broad and include five criteria… 5) knowledge of the COVID-19 vaccine.” Kindly expand on why women with no knowledge of the COVID-19 vaccine will be excluded from the study.
  - If a participant was unaware of the COVID-19 vaccine, they would be unable to answer many questions on the questionnaire. The purpose of the study was to identify attitudes towards COVID-19 vaccination, asking someone who doesn’t know the vaccine exists would not fulfill our study’s objectives.
- Page 7: “…the script also asks for … COVID-19 vaccination status.” The authors should clarify if this will be based on patient recall only or corroborated by valid vaccination records. Where limitations arise with regards to the data source, do ensure that this is appropriately addressed in a study limitations section.
  - We have clarified this in the methods.
- Page 8: “Questions related to the following constructs are included…disease, knowledge of vaccines…” Given that women who demonstrate no or low knowledge of COVID-19 vaccines would be ineligible to participate, how would this component feature in the study?
  - The knowledge questions are not related to whether they know about the vaccine or not, rather, they dive deeper into their understanding of the disease itself, the vaccine, awareness of different brands, and its intended use and recommendations.
- Page 8: “All countries are digitally audio recording… Brazil and Kenya are using paper-based data collection…” The authors should comment on how the diverse data collection formats could influence the accuracy and quality of data collected and the measures to be taken for consolidation of data across participating countries during the analysis stage. In addition, the authors should address how they will treat missing data.
  - We have clarified how data was kept consistent across countries and have added in a statement regarding missing data.
- Page 8: “Both components of the study (questionnaires and interviews) …” RE: Questionnaires, the authors should clarify if there will be back translation.
  - We clarified that all translations were reviewed by a second research team member.
- Page 8: “For the qualitative aim of the study, a grounded theory approach…” Kindly provide a justification for this approach and why it is best suited for this study.
  - We choose grounded theory and thematic analysis to allow for themes to emerge from the empirical data, this has been added to the manuscript.
- The data analysis approach for the quantitative arm of the study is insufficiently addressed. Kindly provide a detailed analysis plan, highlighting any variations across countries / settings.
  - Details of the analyses will be outlined in each of the manuscripts that present the analysis results. We feel this is much better placed alongside the results of the analysis rather than in this protocol paper. We have clarified how statistical tests will be chosen.   
- As part of the “Dissemination plans”, the authors should address how study findings will be shared with participants and immediate affected communities (i.e., what arrangements have been made for community engagement and feedback?).
  - As this study collected no PII, we are unable to share results directly with participants and given that most of the facilities where participants were recruited are referral facilities, the participants came from wide geographical areas making dissemination to the community unrealistic. The dissemination plans were chosen by each research site to be the most appropriate audiences for the results of the research. Most countries are targeting healthcare professionals, which given the attitudinal nature of the data, is likely the most appropriate audience to share results with.
- The authors should consolidate the "Ethical review" and the “Ethics and consent” sections to avoid repetition.
  - This was a requirement of the journal, not a choice by the authors.
- Page 10: “All four countries used written informed consent, using alternatives for illiterate participants…” Kindly specify the alternative approaches adopted for the consenting process for participants who were unable to read or write.
  - This has been added.
- The authors should include a study limitations section, comprehensively addressing all limitations associated with the design, methodological tools and approaches, and execution of this study.
  - Similar to the analysis plans, the limitations directly impact the interpretation of the results, therefore, they will be included in each of the manuscripts that present data.
